# Optimizing Mental Health Act Referrals: A Quality Improvement Project to Enhance Documentation and Ensure Least Restrictive Practice in an Acute Hospital Setting

**DOI:** 10.1192/bjo.2026.11754

**Published:** 2026-06-30

**Authors:** Oluwafisayo Williams, Bushra Azam

**Affiliations:** Derbyshire Healthcare Foundation Trust, Chesterfield, United Kingdom

## Abstract

**Aims:**

Guided by the Model for Improvement, this project aims to increase the completion of core assessments and capacity-to-consent documentation in MHA referrals made by the MHLT to at least95% within six months. The ultimate objective is to enhance resource efficiency, ensure compliance with the MHA Code of Practice, and guarantee that formal detention is initiated only when legally and clinically justified.

**Methods:**

A baseline diagnostic analysis of referrals from the Mental Health Liaison Team (MHLT) at Chesterfield Royal Hospital revealed that 33% (10/30) of MHA referrals did not result in detention. Critically, 90% of patients without a recorded core assessment and 70% without documented capacity were ultimately admitted informally or discharged. This diagnostic phase indicated a direct correlation between missing documentation and a lack of clinical necessity for formal detention, suggesting that a lack of structured assessment acted as a barrier to identifying less restrictive alternatives.

The project involved mapping the current MHLT referral process, identifying a lack of a structured, mandatory protocol as a key driver for poor-quality referrals. A driver diagram was developed to focus on staff competency and referral mechanics. Two PDSA cycles are currently being implemented:

- **System Mechanics:** Introduction of mandatory digital fields in the referral pathway requiring confirmation of a core assessment, capacity assessment, and documented consideration of informal admission.
- **Staff Competency:** Delivery of structured teaching sessions to MHLT staff regarding MHA principles and the introduction of visual aids prompting documentation prior to referral submission.

**Results:**

Based on the diagnostic phase, which showed that formal assessment was more frequently omitted in referrals that ultimately did not require detention, we expect a substantial improvement in referral quality by ensuring these steps are taken pre-referral. We are currently implementing these changes and monitoring process measures. A follow-up audit will be conducted to formally measure the increase in documentation compliance and the subsequent reduction in referrals not resulting in detention, aiming to demonstrate improved resource efficiency and adherence to the MHA Code of Practice.

**Conclusion:**

This ongoing QI initiative indicates that structural changes to referral mechanisms, paired with targeted staff education, can be effective in improving documentation fidelity. By enhancing the decision filter for referrers, this project aims to ensure that MHA assessments are initiated only when legally and clinically justified

